# FOXF1 and alveolar–vascular dysregulation in bronchopulmonary dysplasia–associated pulmonary hypertension

**DOI:** 10.3389/fphys.2026.1910923

**Published:** 2026-10-07

**Authors:** Xinyu Wang, Jun Han, Su Yang, Minghui Xiu, Zekun Yu

**Affiliations:** Department of Neonatology, Children’s Medical Center, The First Hospital of Jilin University, Changchun, China

**Keywords:** alveolar–vascular coupling, angiogenesis, bronchopulmonary dysplasia, FOXF1, lung development, pulmonary hypertension

## Abstract

Bronchopulmonary dysplasia-associated pulmonary hypertension (BPD-PH) is a severe complication of prematurity characterized by arrested alveolarization, pulmonary vascular rarefaction, and progressive vascular remodeling. Increasing evidence suggests that disruption of alveolar–vascular development is a central mechanism underlying disease progression; however, the molecular regulators coordinating these processes remain incompletely understood. Forkhead box F1 (FOXF1) is a developmental transcription factor essential for pulmonary vascular growth, endothelial homeostasis, and alveolar development. Recent studies have identified FOXF1 as a key regulator of endothelial progenitor cell maintenance and alveolar–vascular coupling through interactions with multiple signaling pathways, including VEGF/FLK1, BMP9/ACVRL1, PDGF/TIE2, Notch, WNT5A, and Semaphorin-associated networks. Through these pathways, FOXF1 coordinates angiogenesis, vascular maturation, epithelial differentiation, and tissue repair during lung development. In this review, we summarize the developmental biology of FOXF1 and examine current evidence linking FOXF1 dysregulation to impaired alveolar–vascular development in BPD-PH. We discuss how reduced FOXF1 activity may contribute to endothelial dysfunction, defective angiogenic repair, disrupted epithelial–mesenchymal communication, and pathological vascular remodeling. We further evaluate emerging FOXF1-targeted therapeutic approaches, including nanoparticle-mediated gene delivery, adeno-associated virus (AAV)-based strategies, small-molecule FOXF1 stabilizers, and human vascularized lung organoid platforms. Collectively, available evidence supports a model in which FOXF1 functions as a molecular integrator of alveolar and vascular development, linking arrested alveolarization to pulmonary vascular remodeling in BPD-PH. Nevertheless, current knowledge is derived largely from experimental models and observational human studies. Future investigations integrating human multi-omics datasets, advanced organoid systems, and translational animal models will be essential to determine the causal role of FOXF1 and its potential as a biomarker and therapeutic target in BPD-PH.

## 1 Introduction

Bronchopulmonary dysplasia (BPD) remains one of the most common chronic complications of prematurity and continues to be a major contributor to long-term respiratory morbidity among survivors of extremely preterm birth (Jensen et al., 2019; Sheth et al., 2020; Hansmann et al., 2021). Despite substantial advances in neonatal intensive care, including the widespread use of antenatal corticosteroids, surfactant replacement, and noninvasive respiratory support, the incidence of BPD has not declined significantly in the most immature infants (Thébaud et al., 2019; El-Saie et al., 2023). Among affected infants, pulmonary hypertension (PH) develops in approximately 25–33% of those with moderate-to-severe BPD and is associated with increased mortality, impaired somatic growth, prolonged hospitalization, and adverse cardiopulmonary outcomes extending into childhood and adulthood (Hansmann et al., 2021; El-Saie et al., 2023).

The pathogenesis of BPD-associated pulmonary hypertension (BPD-PH) is complex and multifactorial. Current evidence suggests that pulmonary vascular abnormalities are not merely secondary consequences of lung injury but are central drivers of disease progression (Mourani et al., 2015; Durlak and Thébaud, 2024). Histopathologically, BPD-PH is characterized by arrested alveolarization, pulmonary vascular rarefaction, abnormal muscularization of small pulmonary arteries, and progressive vascular remodeling, ultimately resulting in increased pulmonary vascular resistance and right ventricular dysfunction (Khemani et al., 2007; Hansmann et al., 2021; Durlak and Thébaud, 2024). These observations have led to the “vascular hypothesis” of BPD, which proposes that impaired pulmonary vascular development directly disrupts alveolar growth through the loss of endothelial–epithelial signaling interactions required for normal lung maturation (Durlak and Thébaud, 2024).

Increasing evidence from developmental biology has demonstrated that alveolar and vascular development are tightly coordinated processes during late lung morphogenesis. Formation of the alveolar gas-exchange unit depends on reciprocal communication among epithelial, mesenchymal, endothelial, and perivascular cell populations. Disruption of these interactions not only impairs pulmonary angiogenesis but also arrests secondary septation and alveolar maturation (Abman, 2001; Thébaud and Abman, 2007; Baker and Abman, 2015; Bolte et al., 2017; Ren et al., 2019; Zepp and Morrisey, 2019; Durlak and Thébaud, 2024). Consequently, identifying upstream regulators that coordinate both vascular and alveolar development has become a major focus in efforts to understand the pathobiology of BPD-PH.

Among the candidate regulatory molecules, Forkhead Box F1 (FOXF1) has emerged as a particularly compelling factor because of its essential roles in lung development, pulmonary angiogenesis, endothelial homeostasis, and mesenchymal differentiation (Mahlapuu et al., 2001b; Stankiewicz et al., 2009; Ren et al., 2014). FOXF1 belongs to the forkhead box family of transcription factors and is highly expressed in pulmonary mesenchymal cells, capillary endothelial cells, and pericyte populations during lung development (Stankiewicz et al., 2009; Guo et al., 2023; Shirazi et al., 2025). Experimental studies have demonstrated that FOXF1 regulates multiple signaling pathways involved in vascular growth and tissue morphogenesis, including VEGF/FLK1, BMP9/ACVRL1, PDGF/TIE2, Notch, and WNT signaling networks (Kalinichenko et al., 2004; Ren et al., 2014; Sturtzel et al., 2018; Wang et al., 2022; Reza et al., 2023). Through these regulatory functions, FOXF1 occupies a strategic position at the interface between pulmonary vascular development and alveolar formation.

The biological significance of FOXF1 is further underscored by its established role in alveolar capillary dysplasia with misalignment of pulmonary veins (ACDMPV), a lethal developmental lung disorder caused predominantly by FOXF1 mutations or disruption of its regulatory elements (Stankiewicz et al., 2009; Bishop et al., 2011; Szafranski et al., 2013; Szafranski et al., 2016a; Guo et al., 2023). Although ACDMPV and BPD-PH are distinct clinical entities with different etiologies and developmental trajectories, both disorders share critical features of impaired pulmonary vascular development and defective alveolar–capillary formation. Insights gained from studies of FOXF1-associated developmental lung disease therefore provide a valuable framework for understanding mechanisms that may also contribute to BPD-PH.

More recently, advances in single-cell transcriptomics, spatial transcriptomics, and multi-omics approaches have identified FOXF1-dependent endothelial progenitor populations and revealed previously unrecognized signaling networks linking FOXF1 to pulmonary vascular repair and alveolar maturation (Ren et al., 2019; Wang et al., 2022; Guo et al., 2023; Shirazi et al., 2025). These findings have expanded the role of FOXF1 beyond a developmental transcription factor and suggest that FOXF1 may function as a molecular coordinator of alveolar–vascular coupling in both physiological lung development and pathological lung injury.

In this review, we summarize the developmental biology and regulatory mechanisms of FOXF1, examine current evidence linking FOXF1 dysregulation to BPD-PH, and discuss the signaling pathways through which FOXF1 may influence alveolar–vascular development. We further evaluate emerging translational strategies targeting FOXF1 and propose a conceptual framework in which FOXF1 serves as a molecular nexus linking arrested alveolarization and pulmonary vascular remodeling, the two defining pathological hallmarks of BPD-PH.

## 2 Biological characteristics and developmental regulation of FOXF1

### 2.1 Gene structure and regulatory features of FOXF1

FOXF1 is located on human chromosome 16q24.1 and encodes a transcription factor containing a winged-helix DNA-binding domain; it belongs to the FOX transcription factor superfamily (Mahlapuu et al., 2001b; Stankiewicz et al., 2009). Approximately 270 kb upstream of FOXF1 lies a 60-kb distal lung-specific enhancer region, which contains multiple long non-coding RNA genes and CpG-enriched sites; this region serves as an essential regulatory element for the specific expression of FOXF1 in fetal lung tissue (Szafranski et al., 2016b). Recent single-cell multi-omics studies (combining single-cell RNA sequencing with ATAC sequencing) have identified four evolutionarily conserved endothelial and mesenchymal cell-specific enhancers (FEL-1 to FEL-4) upstream of the FOXF1 locus, elucidating the epigenetic regulatory mechanisms underlying the cell-type-specific expression of FOXF1 (Wang et al., 2024).

At the protein level, FOXF1 is primarily expressed in pulmonary capillary endothelial cells, mesenchymal cells, and pericytes, playing a key role in the formation and maintenance of the alveolar gas exchange unit (Stankiewicz et al., 2009; Guo et al., 2023; Shirazi et al., 2025). In adult lung tissue, FOXF1 is selectively expressed in pulmonary capillary endothelium, while it is scarcely expressed in the endothelium of large pulmonary vessels (pulmonary arteries and veins); this specific expression pattern lays the foundation for the development of pulmonary endothelial-targeted therapeutic strategies (Ren et al., 2014; Isobe et al., 2023).

### 2.2 Expression patterns of FOXF1 during lung development and postnatal homeostasis

During embryonic development, FOXF1 is directly regulated by the Sonic Hedgehog (SHH) signaling pathway. Exogenous SHH activates FOXF1 transcription in the developing fetal lung, whereas in SHH-deficient mice, FOXF1 mRNA expression is completely absent in the lung and foregut mesenchyme (Mahlapuu et al., 2001a; Morrisey and Hogan, 2010; Schittny, 2017). BMP4 can antagonize SHH-mediated activation of FOXF1, while FGF10 and FGF7 can also downregulate FOXF1 expression via distinct mechanisms (Mahlapuu et al., 2001a; Schittny, 2017). Furthermore, FOXF1 directly activates the transcription of multiple Wnt ligands, including Wnt2 and Wnt11, while simultaneously transcribing the Wnt signaling antagonist Wif1, thereby maintaining appropriate Wnt signaling levels to promote lung morphogenesis (Ustiyan et al., 2018). It also regulates the mesenchymal-epithelial crosstalk by activating WNT5A, influencing the differentiation balance between alveolar type I and type II epithelial cells (Reza et al., 2023).

Animal models have revealed the dose-dependent nature of FOXF1: Foxf1 homozygous knockout mice die before embryonic day 10 due to extra-embryonic mesodermal defects; Foxf1 heterozygous knockout mice (Foxf1+/-) exhibit reduced alveolar capillaries and a neonatal mortality rate of approximately 90% (Mahlapuu et al., 2001a). Endothelial-specific FOXF1 knockout (Tie2-Cre Foxf1fl/fl) results in severe defects in yolk sac and placental vasculature, leading to embryonic lethality (Ren et al., 2014). Mesenchymal-specific FOXF1 deficiency disrupts lung morphogenesis, causing abnormalities in lung bud formation and airway branching (Ustiyan et al., 2018).

### 2.3 Maternal nutritional and fetal genetic influences on FOXF1-related lung development

Maternal nutritional status and fetal genetic background may influence pulmonary development by modifying the developmental signaling networks in which FOXF1 functions. Among maternal nutritional factors, vitamin A and its active metabolite, all-trans retinoic acid (ATRA), have attracted particular attention because retinoic acid acts as a stage-dependent morphogen during embryogenesis and is indispensable for early lung specification and morphogenesis (Niederreither et al., 1999; Coste and Labbe, 2011).

Experimental studies have demonstrated that endogenous retinoic acid signaling is required for respiratory field specification, primary lung-bud formation, and early branching morphogenesis. Disruption of retinoic acid synthesis or receptor signaling suppresses FGF10 expression, impairs mesenchymal proliferation, and may result in severe pulmonary hypoplasia or lung agenesis (Malpel et al., 2000; Desai et al., 2004; Chen et al., 2007; Chen et al., 2010). Rather than acting through a single pathway, retinoic acid interacts with multiple developmental signaling cascades, including FGF, SHH, BMP, and WNT, that collectively regulate lung morphogenesis (Malpel et al., 2000; Desai et al., 2004; Chen et al., 2007; Chen et al., 2010). Because SHH is an established upstream regulator of FOXF1 during pulmonary mesenchymal development, alterations in maternal retinoid availability could theoretically influence FOXF1-dependent developmental programs through modification of this broader signaling environment (Malpel et al., 2000; Mahlapuu et al., 2001a; Desai et al., 2004; Chen et al., 2007; Chen et al., 2010). However, direct evidence demonstrating that maternal vitamin A status or maternal ATRA supplementation quantitatively regulates FOXF1 expression in fetal lung tissue remains unavailable.

The biological effects of retinoic acid are highly dependent on developmental timing. During early embryogenesis, insufficient retinoid signaling disrupts lung-bud initiation, whereas excessive or prolonged retinoic acid exposure during branching morphogenesis may interfere with normal airway patterning (Malpel et al., 2000; Desai et al., 2006). Likewise, maternal vitamin A deficiency has been associated with impaired airway branching, abnormal distal airspace formation, reduced elastic fiber deposition, and delayed pulmonary vascular development in experimental models (Antipatis et al., 1998). These observations suggest that both retinoid deficiency and excessive exposure may adversely affect fetal lung development, although their effects on FOXF1 are likely to be indirect.

Fetal genetic factors may further modify the pulmonary response to maternal retinoid signaling. HOXA1 is a retinoic-acid-responsive homeobox transcription factor involved in early embryonic patterning. A recent Hoxa1-null piglet study demonstrated impaired alveolar development and pulmonary microvascular growth, both of which were partially rescued by maternal ATRA administration during early gestation. The rescue was accompanied by increased expression of several angiogenesis-associated genes, including KDR, VEGFD, PDGFD, and ID1 (Chen et al., 2023). However, FOXF1 expression was not examined in that study, and no experimental evidence currently demonstrates direct regulation of the FOXF1 locus by HOXA1.

Collectively, available evidence supports the concept that maternal retinoid signaling and fetal genetic background influence the broader developmental environment in which FOXF1 functions rather than directly regulating FOXF1 itself. Future studies integrating fetal lung transcriptomics, chromatin-accessibility profiling, and conditional developmental models will be required to determine whether these maternal and fetal factors directly regulate FOXF1 or primarily influence parallel signaling pathways involved in alveolar–vascular development.

### 2.4 Dose-dependent effects of FOXF1 during pulmonary development

FOXF1 is a dosage-sensitive developmental transcription factor that is indispensable for normal pulmonary vascular and alveolar development. However, no quantitative physiological concentration range has yet been established for FOXF1 during fetal lung development. Existing studies have primarily evaluated relative gene dosage, mRNA expression, or protein abundance in genetic and experimental models rather than defining an absolute concentration threshold. Moreover, because FOXF1 expression varies according to developmental stage, pulmonary cell type, and anatomical compartment, a universal physiological concentration is unlikely to be applicable across different stages of lung development (Kalinichenko et al., 2001; Mahlapuu et al., 2001a; Ren et al., 2014; Ustiyan et al., 2018; Wang et al., 2022).

Likewise, the developmental consequences of sustained FOXF1 overexpression remain poorly understood. Current gene-delivery and small-molecule studies have largely focused on restoring reduced FOXF1 activity in disease or injury models rather than inducing supraphysiological FOXF1 expression in healthy fetal lungs (Bolte et al., 2020; Kohram et al., 2023; Pradhan et al., 2023). Although preliminary studies have not reported overt short-term toxicity following FOXF1 restoration in wild-type mice, the long-term developmental safety of persistent FOXF1 activation has not been systematically evaluated (Kohram et al., 2023).

From a developmental perspective, excessive FOXF1 activity could theoretically disturb the balance between endothelial proliferation and differentiation, alter pulmonary vascular maturation, or disrupt epithelial–mesenchymal communication required for normal alveolarization. Nevertheless, these possibilities remain biologically plausible rather than experimentally established and should therefore be interpreted cautiously (Ren et al., 2014; Ustiyan et al., 2018; Wang et al., 2022; Reza et al., 2023).

Future studies using inducible, cell-type-specific FOXF1 overexpression models will be required to define developmental stage-specific dose–response relationships and establish safe therapeutic windows for FOXF1-targeted interventions.

## 3 FOXF1-regulated signaling networks in physiological alveolar–vascular development

Normal lung development requires tightly coordinated interactions among epithelial, endothelial, mesenchymal, and perivascular cell populations. Rather than acting through a single downstream pathway, FOXF1 functions as a transcriptional hub that integrates multiple signaling networks involved in endothelial progenitor cell maintenance, vascular growth, vessel maturation, epithelial differentiation, and tissue repair (Morrisey and Hogan, 2010; Ren et al., 2014; Sturtzel et al., 2018; Ren et al., 2019; Wang et al., 2022; Reza et al., 2023). Emerging evidence suggests that disruption of FOXF1 signaling simultaneously affects several interconnected developmental pathways, ultimately leading to impaired alveolarization and pulmonary vascular rarefaction (Ren et al., 2014; Ren et al., 2019; Wang et al., 2022; Bian et al., 2023). The major FOXF1-dependent signaling networks identified to date include VEGF/FLK1, BMP9/ACVRL1, PDGF/TIE2, Notch, WNT5A, and Semaphorin-associated pathways, which collectively regulate alveolar–vascular development and homeostasis (Kalinichenko et al., 2004; Ren et al., 2014; Sturtzel et al., 2018; Wang et al., 2022; Reza et al., 2023; Shirazi et al., 2025).

### 3.1 VEGF/FLK1 signaling and embryonic angiogenesis

The earliest and most fundamental role of FOXF1 in vascular development is the transcriptional regulation of vascular endothelial growth factor (VEGF) receptor expression. Foxf1 +/− mice exhibit defects in alveolarization and angiogenesis, and the pulmonary malformations associated with the Foxf1 +/− mutation are accompanied by reduced expression of vascular endothelial growth factor (VEGF) and VEGF receptor 2 (Flk-1) (Kalinichenko et al., 2001). Conditional knockout of Foxf1 in endothelial cells using the Tie2-Cre and Pdgfb-CreER transgenic systems results in embryonic lethality, accompanied by the disappearance of FLK1 (VEGFR2/KDR) and FLT1 (VEGFR1) from the endothelial cell surface, accompanied by growth retardation, polyhydramnios, ventricular hypoplasia, and severe vascular malformations in the lungs, placenta, yolk sac, and retina (Ren et al., 2014; Cai et al., 2016).

In the neonatal lung, impaired downstream VEGF receptor signaling following FOXF1 deletion directly leads to reduced alveolar capillary density. There is an interaction between the alveolar capillary bed and developing epithelial cells: VEGF secreted by alveolar type I (AT1) cells promotes endothelial cell proliferation, while trophic signals released by endothelial cells (including epidermal growth factor (EGF) ligands mediated by matrix metalloproteinase 14 (MMP14)) support epithelial cell maturation (Bolte et al., 2017; Ren et al., 2019). The loss of FOXF1 disrupts this vascular-epithelial axis, thereby impairing alveolar capillary formation and the alveolar septum formation process it supports.

Collectively, these findings position the FOXF1–VEGF axis as a fundamental regulator of alveolar–vascular coupling. By maintaining endothelial responsiveness to VEGF signaling, FOXF1 supports not only pulmonary angiogenesis but also the reciprocal endothelial–epithelial interactions required for normal alveolar septation and maturation (Kalinichenko et al., 2001; Ren et al., 2014; Bolte et al., 2017; Ren et al., 2019).

### 3.2 The BMP9/ACVRL1/SMAD1 pathway in neonatal pulmonary angiogenesis

Using single-cell RNA sequencing (scRNA-seq), Wang et al. revealed that pulmonary endothelial progenitor cells (EPCs) exhibit distinctive characteristics of the BMP9/ACVRL1/SMAD1 transcriptional pathway, which is largely dependent on FOXF1 (Wang et al., 2022). ACVRL1 (activin receptor-like kinase 1, also known as ALK1) is the primary BMP9 receptor on endothelial cells and a well-established driver of pulmonary vascular development (Trembath et al., 2001). FOXF1 acts in synergy with the ETS family transcription factor FLI1 to directly activate the ACVRL1 promoter: these two proteins jointly bind to complex regulatory elements within the ACVRL1 promoter, while inhibition of FOXF1 or deletion of FLI1 reduces ACVRL1 transcription levels and impairs *in vitro* angiogenesis (Wang et al., 2022).

In wild-type neonatal mice, nanoparticle-mediated ACVRL1 silencing resulted in a BPD-like phenotype, including reduced alveolar angiogenesis and impaired alveolarization, thereby establishing ACVRL1 as a pathogenic mediator of impaired lung development rather than a mere associate (Wang et al., 2022). Consequently, the FOXF1-FLI1-ACVRL1 axis constitutes a critical pro-angiogenic signaling cascade, the disruption of which contributes to impaired neonatal pulmonary angiogenesis and a BPD-like developmental phenotype, with potential relevance to BPD-PH.

Importantly, the FOXF1–FLI1–ACVRL1 signaling cascade represents one of the strongest mechanistic links between FOXF1 deficiency and impaired neonatal lung angiogenesis. Unlike pathways inferred primarily from association studies, disruption of ACVRL1 signaling has been experimentally demonstrated to induce a BPD-like phenotype, supporting a causal role for this pathway in developmental lung injury (Trembath et al., 2001; Wang et al., 2022).

### 3.3 FOXF1/R-Ras signaling and vascular homeostasis

The FOXF1 protein, acting as an anti-fibrotic factor, regulates fibroblast function both *in vitro* and *in vivo* (Black et al., 2018). FOXF1 has been identified as one of the endothelial transcription factors exhibiting the most significant dysregulation during the fibrotic process, and the extent of FOXF1 downregulation correlates with increasing disease severity (Bian et al., 2023). In mice, endothelial-specific inhibition of Foxf1 increased collagen deposition, promoted pulmonary inflammation, and impaired R-Ras-dependent vascular normalization signaling; *in vitro* studies demonstrated that endothelial cells lacking FOXF1 exhibited enhanced proliferation and invasiveness, and could activate human pulmonary fibroblasts via paracrine mechanisms, stimulating macrophage migration through their pro-inflammatory secretome (Bian et al., 2023). These findings extend the scope of FOXF1’s role from angiogenesis to broader pathological vascular-stromal remodeling, encompassing the pathological processes underlying severe bronchopulmonary dysplasia (BPD) and its transition to BPD-PH.

Emerging evidence suggests that endothelial progenitor cells (EPCs) represent an important cellular component of FOXF1-mediated vascular repair and regeneration. EPCs contribute to postnatal pulmonary angiogenesis by supporting endothelial renewal, vascular growth, and microvascular maintenance during lung development. Clinical studies have demonstrated alterations in circulating EPC populations in preterm infants who subsequently develop BPD, suggesting that impaired endothelial regenerative capacity may contribute to disease susceptibility (Baker et al., 2009; Borghesi et al., 2009).

Experimental studies further support a functional role for EPCs in developmental lung injury. In a hyperoxia-induced model of arrested alveolar growth, endothelial colony-forming cells (ECFCs), a highly proliferative EPC subtype, exhibited impaired angiogenic function, whereas restoration of ECFC activity improved pulmonary vascular growth and alveolar development (Alphonse et al., 2014). Given the established role of FOXF1 in maintaining endothelial identity and angiogenic signaling, disruption of FOXF1 activity may compromise EPC-mediated vascular regeneration and thereby contribute to impaired alveolar–vascular development.

These observations suggest that FOXF1 contributes not only to vascular formation during development but also to the maintenance of endothelial stability after injury. Consequently, FOXF1 deficiency may promote a transition from adaptive vascular remodeling to pathological fibroproliferative remodeling, a process potentially relevant to severe BPD and BPD-PH (Black et al., 2018; Bian et al., 2023).

### 3.4 FOXF1 regulation of vascular maturation and stabilization

Beyond its role in endothelial proliferation, FOXF1 also contributes to later stages of vascular development by regulating pathways involved in vessel maturation and stabilization. Among these, PDGF/TIE2 signaling promotes pericyte recruitment and vascular integrity, whereas Notch signaling regulates endothelial specification, vascular branching, and endothelial progenitor cell function (Suri et al., 1996; Hellström et al., 1999; Kalinichenko et al., 2004; Gridley, 2007; Ren et al., 2014; Sturtzel et al., 2018).

FOXF1 is also associated with signaling pathways involved in pulmonary vascular development, including the Notch and PDGF/TIE2 networks. Experimental studies have shown that FOXF1 regulates the expression of PDGFB and TIE2, contributing to pericytic recruitment and vascular stabilization; FOXF1 deficiency leads to downregulation of PDGFB and TIE2, reduced pericytic coverage, and impaired vascular maturation (Ren et al., 2014). Concurrently, FOXF1 deficiency leads to downregulation of Notch-2 signaling, inhibiting the sprouting capacity of endothelial colony-forming cells (ECFCs) and causing abnormalities in microvascular development (Kalinichenko et al., 2004; Sturtzel et al., 2018).

Therefore, FOXF1 appears to coordinate multiple stages of vascular development, ranging from endothelial progenitor expansion to vascular stabilization and maturation. This broad regulatory capacity may explain why FOXF1 deficiency produces complex developmental phenotypes that cannot be attributed to disruption of a single downstream pathway (Kalinichenko et al., 2004; Ren et al., 2014; Sturtzel et al., 2018; Wang et al., 2022).

Taken together, these findings indicate that FOXF1 regulates multiple stages of alveolar–vascular development through a coordinated network of signaling pathways rather than through a single downstream effector, providing the basis for the integrated physiological framework proposed below.

### 3.5 An integrated physiological conceptual framework of FOXF1-dependent alveolar–vascular development

Although individual FOXF1-regulated signaling pathways have often been investigated separately, increasing evidence indicates that they function as components of an interconnected developmental network rather than as independent molecular cascades. Therefore, we propose an integrated physiological conceptual framework of FOXF1-dependent alveolar–vascular development (**Figure 1**). This framework synthesizes the coordinated regulation of VEGF/FLK1-mediated angiogenesis, BMP9/ACVRL1-dependent endothelial progenitor cell maintenance, PDGFB/TIE2-mediated vascular maturation, Notch-regulated endothelial sprouting, and WNT5A-dependent epithelial differentiation into a unified model governing pulmonary alveolar–vascular coupling (Kalinichenko et al., 2004; Ren et al., 2014; Sturtzel et al., 2018; Zepp and Morrisey, 2019; Wang et al., 2022; Reza et al., 2023).

**Figure 1:**
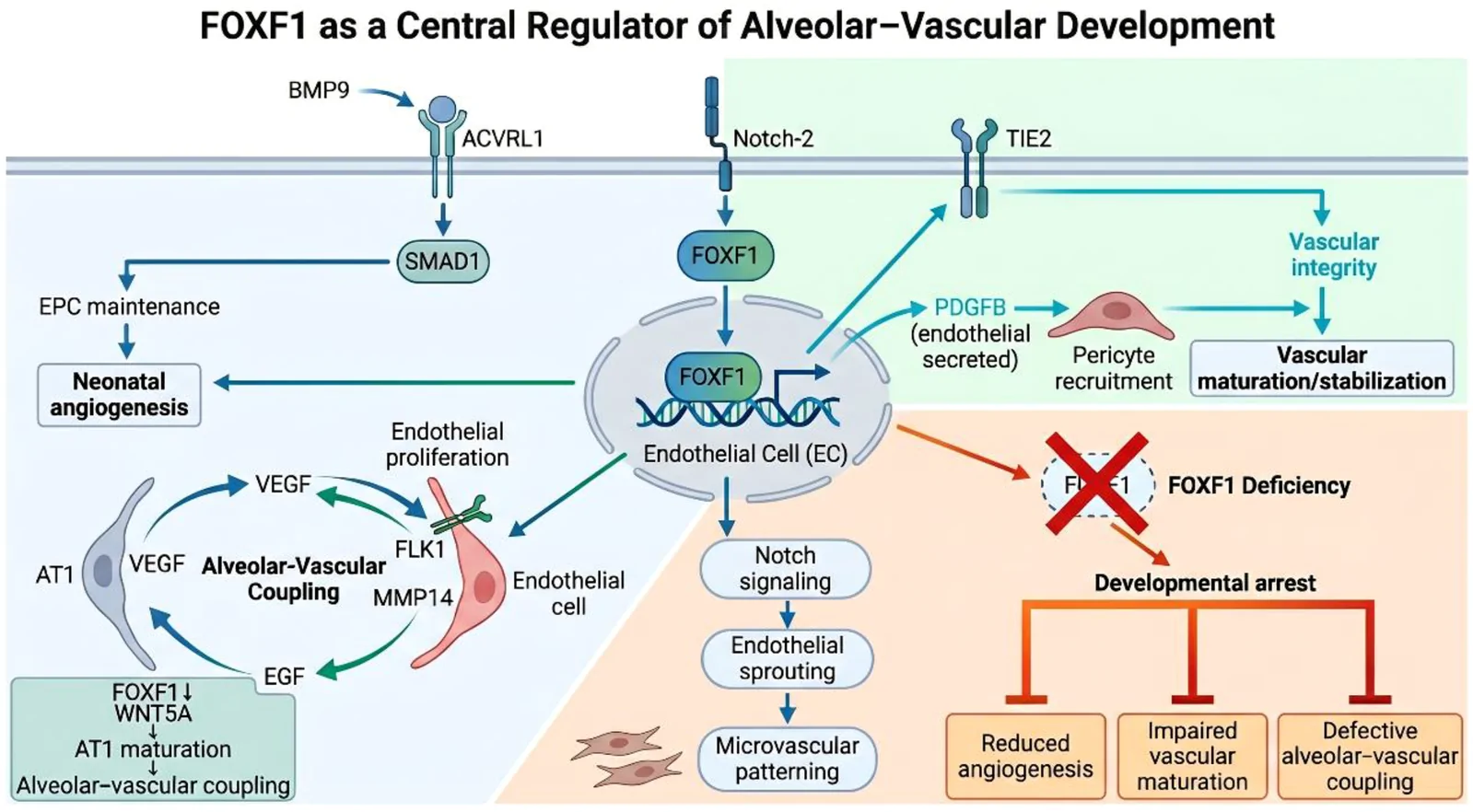
FOXF1 functions as a transcriptional regulator in pulmonary endothelial cells and coordinates multiple signaling pathways involved in alveolar–vascular development. FOXF1 promotes endothelial progenitor cell (EPC) maintenance and neonatal angiogenesis through interactions with the BMP9/ACVRL1/SMAD1 pathway and supports endothelial proliferation through VEGF/FLK1 signaling. FOXF1 also contributes to vascular maturation and stabilization by regulating PDGFB-mediated pericyte recruitment and TIE2-associated vascular integrity, while promoting endothelial sprouting and microvascular patterning through Notch signaling. In addition, FOXF1-dependent WNT5A signaling supports alveolar type I (AT1) cell maturation and epithelial–endothelial communication required for alveolar–vascular coupling. FOXF1 deficiency disrupts these coordinated developmental programs, resulting in reduced angiogenesis, impaired vascular maturation, and defective alveolar–vascular coupling. **Figure 1** summarizes the integrated physiological conceptual framework described in Section 3.5 and provides the mechanistic foundation for the disease-oriented conceptual framework illustrated in **Figure 2**.

Within this framework, FOXF1 occupies an upstream regulatory position and functions as a central transcriptional coordinator of endothelial, epithelial, and mesenchymal interactions during lung development (Ren et al., 2014; Wang et al., 2022). **Figure 1** provides a schematic representation of this physiological conceptual framework, illustrating how these interconnected signaling pathways collectively regulate pulmonary vascular growth, alveolar maturation, and epithelial–vascular communication, rather than depicting every molecular interaction individually. Accordingly, **Figure 1** should be interpreted as a conceptual synthesis of the principal physiological signaling pathways discussed in Sections 3.1–3.4, rather than as an exhaustive signaling map.

This integrated physiological framework also provides the mechanistic basis for understanding disease pathogenesis. Rather than affecting a single biological process, reduced FOXF1 activity may simultaneously impair endothelial regeneration, attenuate angiogenic signaling, disrupt epithelial–mesenchymal communication, and compromise vascular maturation, collectively leading to arrested alveolarization and pulmonary vascular remodeling (Ren et al., 2014; Ren et al., 2019; Wang et al., 2022; Bian et al., 2023; Reza et al., 2023; Shirazi et al., 2025). These coordinated pathological alterations are further integrated into the disease-oriented conceptual framework presented in **Figure 2**.

**Figure 2:**
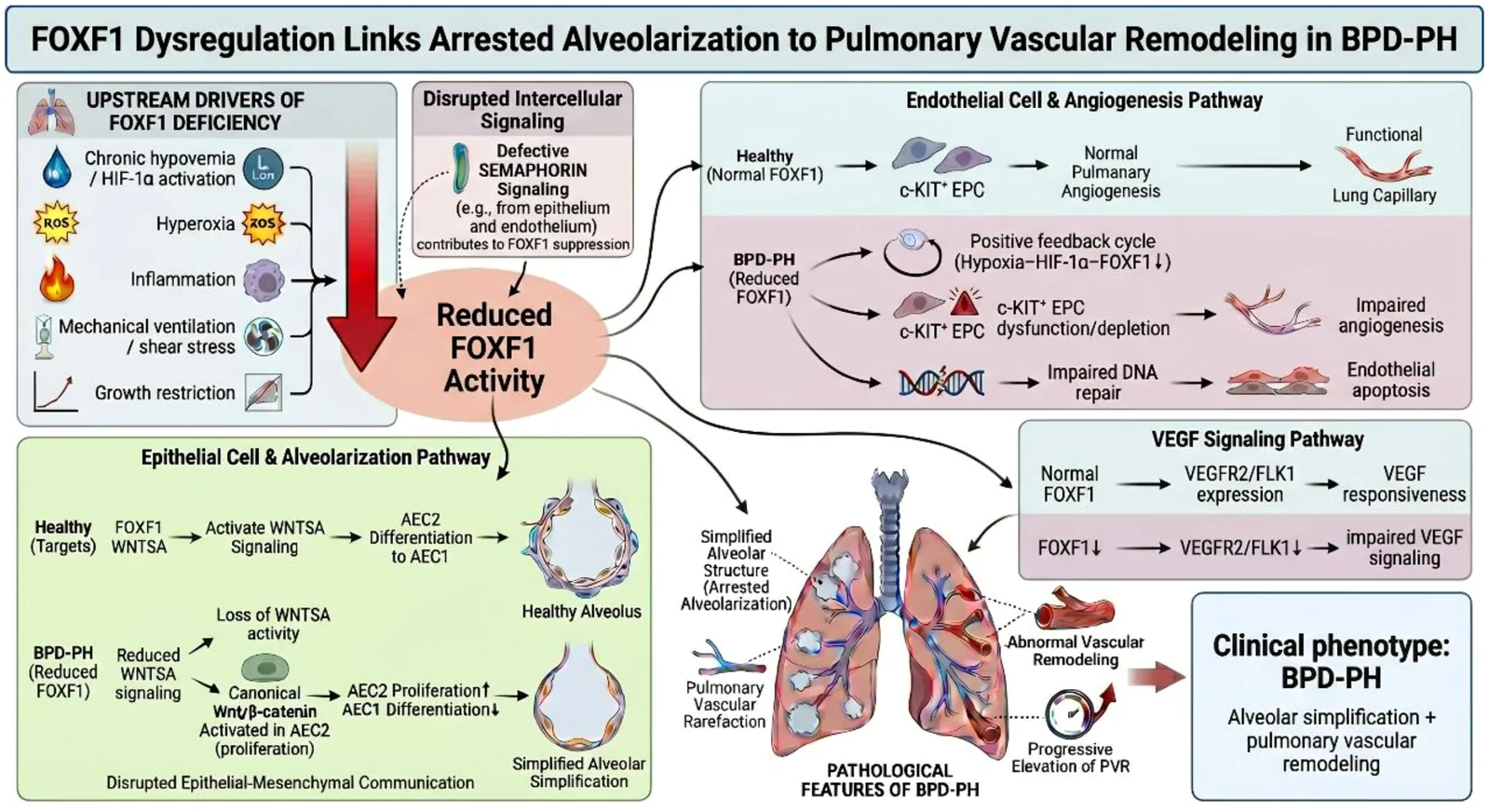
Multiple injurious stimuli associated with prematurity, including hyperoxia, chronic hypoxemia, inflammation, mechanical ventilation, and growth restriction, may converge on FOXF1 downregulation. Loss of FOXF1 activity disrupts endothelial progenitor cell maintenance, impairs angiogenesis, reduces DNA repair capacity, and attenuates VEGF responsiveness through decreased VEGFR2/FLK1 expression. In parallel, disrupted Semaphorin signaling and reduced WNT5A-dependent epithelial–mesenchymal communication impair alveolar epithelial differentiation and alveolar maturation. These convergent abnormalities lead to pulmonary vascular rarefaction, simplified alveolar structure, abnormal vascular remodeling, and progressive elevation of pulmonary vascular resistance, thereby contributing to the development of BPD-PH. BPD-PH, bronchopulmonary dysplasia-associated pulmonary hypertension; EPC, endothelial progenitor cell; PVR, pulmonary vascular resistance; ROS, reactive oxygen species. **Figure 2** illustrates the pathophysiological conceptual framework resulting from disruption of the physiological FOXF1 regulatory network summarized in **Figure 1**.

Given the complexity of the FOXF1 regulatory network, a centralized summary of the principal upstream regulators and downstream effectors is provided in **Table 1**. Importantly, these interactions differ in the strength of available evidence. Some molecules, such as SHH–GLI signaling and VEGFR2/FLK1, have been demonstrated to directly regulate or be regulated by FOXF1 through genetic or transcriptional studies, whereas others, including retinoic acid, BMP signaling, and Semaphorin pathways, primarily exhibit functional interactions within broader developmental signaling networks.

**Table 1:** Integrated regulatory network of FOXF1 during pulmonary alveolar and vascular development.

Regulatory level	Molecule/Gene	Relationship with FOXF1	Biological function	Developmental stage	Evidence strength	Key references
Maternal regulation	Retinoic acid	Indirect developmental influence on the FOXF1-associated signaling environment	Lung bud initiation	Embryonic	★★★☆☆	(Antipatis et al., 1998; Malpel et al., 2000; Desai et al., 2004; Desai et al., 2006; Chen et al., 2007; Chen et al., 2010)
Direct upstream regulators	SHH	SHH-dependent upstream activator	Mesenchymal specification	Embryonic	★★★★★	(Mahlapuu et al., 2001a)
	GLI1/2	Putative mediators of SHH-dependent FOXF1 regulation	FOXF1 transcription	Embryonic	★★★☆☆	(Mahlapuu et al., 2001a; Morrisey and Hogan, 2010)
Developmental signaling interactions	FGF10	Functional interaction	Branching morphogenesis	Embryonic	★★★☆☆	(Malpel et al., 2000; Mahlapuu et al., 2001a; Desai et al., 2004)
	BMP9	Functional interaction	Endothelial differentiation	Fetal/Postnatal	★★★★☆	(Wang et al., 2022)
	HOXA1	Putative indirect regulator	Embryonic patterning	Embryonic	★★☆☆☆	(Langston and Gudas, 1992; Chen et al., 2023)
Environmental regulation	Hyperoxia	FOXF1 suppression	Oxidative injury	Postnatal	★★★★☆	(Bolte et al., 2020)
Downstream targets and FOXF1-associated effectors	VEGFR2 (FLK1/KDR)	Direct target	Endothelial proliferation	Fetal/Postnatal	★★★★★	(Ren et al., 2014; Sturtzel et al., 2018; Wang et al., 2022)
	FLT1	Downstream target	Angiogenesis	Fetal	★★★★☆	(Ren et al., 2014)
	ACVRL1	FOXF1–FLI1 target	EPC maintenance	Postnatal	★★★★★	(Wang et al., 2022)
	PDGFB	Downstream target	Pericyte recruitment	Postnatal	★★★★☆	(Hellström et al., 1999; Ren et al., 2014)
	TIE2	Functional pathway	Vascular maturation	Postnatal	★★★★☆	(Suri et al., 1996; Ren et al., 2014)
	NOTCH2	Functional pathway	Endothelial sprouting	Postnatal	★★★★☆	(Kalinichenko et al., 2004; Sturtzel et al., 2018)
	WNT5A	FOXF1-associated pathway	AEC2→AEC1 differentiation	Alveolar	★★★★☆	(Reza et al., 2023)

Evidence strength was assigned according to the available experimental evidence. ★★★★★, direct genetic, transcriptional, or mechanistic evidence; ★★★★☆, strong functional evidence; ★★★☆☆, moderate experimental evidence; ★★☆☆☆, limited or indirect evidence. Direct regulation refers to experimentally validated transcriptional or chromatin-binding interactions, whereas functional interaction indicates pathway-level regulation without confirmed direct transcriptional binding. Evidence grading reflects the relationship with the broader FOXF1-associated developmental network, not necessarily direct regulation of FOXF1 expression.

## 4 FOXF1 dysregulation in pathological alveolar–vascular development

The defining pathological features of BPD-PH include arrested alveolarization, pulmonary vascular rarefaction, abnormal vascular remodeling, and progressive elevation of pulmonary vascular resistance (Hansmann et al., 2021; El-Saie et al., 2023; Durlak and Thébaud, 2024). Traditionally, these abnormalities have been studied as distinct pathological processes. However, increasing evidence suggests that impaired alveolar development and pulmonary vascular disease are mechanistically interconnected through disruption of alveolar–vascular coupling (Bolte et al., 2017; Ren et al., 2019; El-Saie et al., 2023; Durlak and Thébaud, 2024).

Given its established roles in endothelial progenitor cell maintenance, pulmonary angiogenesis, epithelial differentiation, and vascular maturation, FOXF1 is uniquely positioned to coordinate these developmental processes (Ren et al., 2014; Sturtzel et al., 2018; Ren et al., 2019; Wang et al., 2022; Reza et al., 2023). Emerging experimental and human transcriptomic data support a model in which FOXF1 deficiency contributes simultaneously to endothelial dysfunction, impaired vascular regeneration, disrupted epithelial–mesenchymal communication, and pathological vascular remodeling (Ren et al., 2019; Acharya et al., 2023; Bian et al., 2023; Reza et al., 2023; Shirazi et al., 2025). Consequently, FOXF1 may represent a mechanistic bridge linking arrested alveolarization to pulmonary hypertension in BPD.

### 4.1 Insights from alveolar capillary dysplasia with misalignment of pulmonary veins

Alveolar capillary dysplasia with misalignment of pulmonary veins (ACDMPV) is a rare and usually lethal developmental lung disorder characterized by severe hypoxemia, refractory pulmonary hypertension, and profound abnormalities of pulmonary vascular development shortly after birth (Bishop et al., 2011). Genetic studies have established FOXF1 as the principal disease-causing gene, with most patients carrying heterozygous FOXF1 mutations or disruptions of FOXF1 regulatory elements (Stankiewicz et al., 2009; Szafranski et al., 2013; Szafranski et al., 2016a).

The pathological features of ACDMPV highlight the essential role of FOXF1 in alveolar–vascular development. FOXF1 deficiency disrupts endothelial progenitor cell differentiation, impairs pulmonary capillary formation, and alters signaling interactions between endothelial and alveolar epithelial cells that are required for normal gas-exchange unit development (Guo et al., 2023). Single-cell multi-omics studies have further demonstrated severe depletion of specialized alveolar capillary endothelial populations and widespread abnormalities in vascular developmental programs in FOXF1-deficient lungs (Guo et al., 2023).

Importantly, ACDMPV and BPD-PH differ substantially in their etiology, developmental timing, and clinical manifestations. Therefore, findings derived from ACDMPV should not be directly extrapolated to BPD-PH (Hansmann et al., 2021; El-Saie et al., 2023; Durlak and Thébaud, 2024). Nevertheless, the profound pulmonary vascular defects observed in FOXF1-deficient lungs provide compelling evidence that FOXF1 is indispensable for normal alveolar–vascular development and support investigation of FOXF1-related mechanisms in acquired developmental lung diseases such as BPD-PH (Stankiewicz et al., 2009; Bishop et al., 2011; Szafranski et al., 2013; Szafranski et al., 2016a; Guo et al., 2023).

Although both ACDMPV and BPD-PH are associated with impaired FOXF1-related signaling, their pathogenic consequences differ substantially because FOXF1 dysfunction occurs at distinct stages of lung development. In ACDMPV, germline pathogenic FOXF1 variants exert their effects during embryonic lung organogenesis, when FOXF1 is indispensable for pulmonary organogenesis, including lung-bud formation, pulmonary vascular patterning, and mesenchymal differentiation. Consequently, severe FOXF1 deficiency results in irreversible congenital malformations, including absent alveolar capillaries, misaligned pulmonary veins, and profound pulmonary hypertension that is typically fatal shortly after birth (Ren et al., 2014; Guo et al., 2023; Shirazi et al., 2025).

In contrast, BPD-PH develops after preterm birth, when early lung specification and much of branching morphogenesis have occurred, but distal air-space formation, microvascular expansion, and alveolarization remain highly immature. Rather than causing congenital structural defects, postnatal insults such as hyperoxia, chronic hypoxia, inflammation, and mechanical ventilation may converge on reduced FOXF1-associated signaling within a partially developed lung. As a result, FOXF1 dysregulation primarily impairs endothelial repair, angiogenic maintenance, vascular maturation, and secondary alveolarization, leading to pulmonary vascular rarefaction and simplified alveolar architecture rather than developmental aplasia (Ren et al., 2019; Bolte et al., 2020; Acharya et al., 2023; Durlak and Thébaud, 2024; Shirazi et al., 2025).

These observations suggest that the developmental timing of FOXF1 dysregulation is a major determinant of disease phenotype. Embryonic FOXF1 deficiency disrupts lung construction, whereas postnatal FOXF1 suppression primarily compromises lung maturation and repair. This distinction also provides the biological rationale for FOXF1-directed regenerative therapies in BPD-PH, where residual developmental plasticity may still permit recovery of alveolar and vascular growth. The major distinctions between ACDMPV and BPD-PH are summarized in **Table 2**.

**Table 2:** Developmental and pathological distinctions between ACDMPV and BPD-PH.

Feature	ACDMPV	BPD-PH
Timing	Embryonic	Postnatal
FOXF1 abnormality	Germline mutation	Secondary downregulation
Development	Organogenesis	Maturation
Pathology	Capillary dysplasia	Vascular rarefaction
Reversibility	Minimal	Potentially reversible

### 4.2 Evidence supporting a pathogenic role of FOXF1 dysregulation in BPD-PH

Whether FOXF1 dysregulation acts as a driver of disease pathogenesis or merely reflects ongoing tissue injury remains an important question in BPD-PH research. Several lines of evidence support a potentially pathogenic role for FOXF1 deficiency. First, FOXF1 expression is reduced in experimental models of developmental lung injury and in human BPD lung tissues (Ren et al., 2019; Bolte et al., 2020). Second, genetic or functional suppression of FOXF1 impairs endothelial progenitor cell function, pulmonary angiogenesis, and alveolar development (Ren et al., 2014; Ren et al., 2019; Wang et al., 2022). Third, restoration of FOXF1 or FOXF1-associated signaling through AAV-based gene delivery, nanoparticle approaches, small-molecule stabilization, or downstream STAT3 delivery improves vascular or lung phenotypes in experimental models (Bolte et al., 2020; Sun et al., 2021; Isobe et al., 2023; Kohram et al., 2023; Pradhan et al., 2023). Collectively, these observations suggest that FOXF1 dysregulation may contribute directly to disease progression rather than serving solely as a marker of tissue injury.

FOXF1 deficiency directly impairs the angiogenic capacity of c-KIT+ endothelial progenitor cells. Ren et al. found that FOXF1 and c-KIT are both downregulated in the lung tissue of children with BPD; the transcriptional profile of c-KIT+ endothelial progenitor cells is highly conserved in both mouse and human lungs and is rich in FOXF1-regulated target genes, suggesting that FOXF1 is crucial for maintaining the integrity of the endothelial progenitor cell pool (Ren et al., 2019). Hyperoxic injury reduces FOXF1-associated regenerative capacity in the developing lung, whereas restoration of FOXF1 signaling during the recovery phase promotes endothelial proliferation, angiogenesis, and alveolarization (Bolte et al., 2020). Separately, hypoxia can suppress endothelial FOXF1 through HIF-1α-dependent mechanisms (Acharya et al., 2023). Taken together, these findings support FOXF1-dependent endothelial progenitor cell dysfunction as a plausible mechanism linking developmental lung injury to impaired vascular regeneration. Loss of this regenerative endothelial compartment may contribute to the pathogenesis of BPD-PH (Ren et al., 2019; Shirazi et al., 2025).

Acharya et al. were the first to systematically elucidate the molecular mechanisms linking hypoxia to FOXF1 suppression in pulmonary endothelial cells (Acharya et al., 2023). This finding has profound clinical implications for BPD-PH: chronic hypoxemia—a hallmark of BPD-PH—is expected to maintain the activation of HIF-1α in pulmonary endothelial cells, thereby persistently suppressing FOXF1, impeding angiogenic repair, and exacerbating pulmonary vascular disease. This observation provides a plausible mechanistic explanation for the persistence of vascular abnormalities in BPD-PH. Chronic hypoxemia, a common feature of severe BPD, may perpetuate FOXF1 suppression through sustained HIF-1α activation, thereby creating a self-reinforcing cycle of impaired vascular repair and progressive pulmonary vascular disease (Hansmann et al., 2021; Acharya et al., 2023; El-Saie et al., 2023).

FOXF1 deficiency further exacerbates endothelial dysfunction by impairing DNA repair mechanisms. Isobe et al. found that FOXF1 deficiency reduces the DNA repair capacity of endothelial cells; acting in concert with oxidative stress on top of BMPR2 defects, this accelerates the accumulation of DNA damage, triggers endothelial cell apoptosis and loss of function, and drives the continued progression of pulmonary hypertension (Isobe et al., 2023). Beyond developmental regulation, FOXF1 may also contribute to endothelial resilience under conditions of oxidative and inflammatory stress. This emerging role expands the significance of FOXF1 from a developmental regulator to a potential mediator of vascular repair capacity, which may be particularly relevant in premature lungs exposed to prolonged oxygen therapy and inflammation (Isobe et al., 2023). FOXF1 expression and regulatory activity have been evaluated in human fetal lung tissue and fetal-lung-derived datasets, particularly in studies of ACDMPV and developmental FOXF1 enhancers (Stankiewicz et al., 2009; Szafranski et al., 2016b; Guo et al., 2023; Wang et al., 2024). In contrast, we did not identify published studies that directly quantified FOXF1 expression in human umbilical cord blood.

### 4.3 Semaphorin–FOXF1 signaling: a newly identified pathway in human BPD-PH

Shirazi et al. identified an aberrant capillary cell subpopulation (abCaps) predominantly present in the lung tissue of patients with BPD-PH through endothelial cell analysis, defined by the specific overexpression of ANKRD1 (ankyrin repeat domain 1) (Shirazi et al., 2025). ANKRD1 is a protein associated with mechanical stress responses (Zou et al., 1997); its aberrant expression in BPD-PH endothelial cells suggests that these cells are under persistent mechanical or metabolic stress.

Using CellChat analysis, significant defects were observed in the Semaphorin (Sema)-guided molecular signaling pathways in lung tissue from patients with BPD and BPD-PH, accompanied by persistent downregulation of the pro-angiogenic transcription factor FOXF1 (Shirazi et al., 2025). In BPD-PH, the expression of the Semaphorin ligand-receptor signaling axis is significantly impaired; this Semaphorin signaling defect is associated with functional suppression of FOXF1, disrupting the genetic programs essential for endothelial cell survival and proliferation; Specifically, the absence of Semaphorin signaling is evident in both alveolar epithelial cells and endothelial cells, and has been precisely localized by spatial transcriptomics to developing alveolar regions rather than proximal lung tissue (Shirazi et al., 2025). This signaling abnormality was observed in both hyperoxia-induced BPD models and ACDMPV tissues, suggesting that dysregulated Semaphorin–FOXF1 signaling may represent a shared molecular feature of impaired alveolar development. However, its causal contribution to BPD-PH remains to be established in functional experimental models.

Importantly, the Semaphorin–FOXF1 axis provides one of the first mechanistic explanations linking defective epithelial–endothelial communication to FOXF1 suppression in human BPD-PH. Unlike many previously proposed pathways derived primarily from animal models, these observations were supported by human single-cell and spatial transcriptomic datasets, substantially strengthening their translational relevance (Shirazi et al., 2025). Consequently, Semaphorin signaling may represent an upstream regulatory mechanism through which developmental lung injury converges on FOXF1-dependent endothelial dysfunction.

### 4.4 WNT5A and alveolar epithelial differentiation

FOXF1 regulates mesenchymal-epithelial crosstalk in the pulmonary stroma by transcriptionally activating WNT5A (a non-canonical Wnt ligand), thereby influencing the differentiation of alveolar type 2 epithelial cells (AEC2) and the generation of alveolar type 1 epithelial cells (AEC1) (Reza et al., 2023). Using mouse lung organoids, Reza et al. demonstrated that the S52F FOXF1 mutation activates the canonical Wnt/β-catenin signaling pathway in AEC2, promoting AEC2 proliferation but inhibiting their differentiation into AEC1 (Reza et al., 2023), suggesting a potential mechanism through which FOXF1 dysregulation may contribute to impaired alveolar epithelial differentiation and alveolar simplification in BPD-PH.

These findings suggest that FOXF1 may influence BPD-PH not only through vascular mechanisms but also through direct regulation of alveolar epithelial maturation. Such dual effects further support the concept that FOXF1 occupies a central position within the alveolar–vascular developmental axis (Reza et al., 2023).

### 4.5 FOXF1 dysregulation and impaired VEGF signaling in BPD-PH

VEGF signaling is a central regulator of pulmonary angiogenesis and alveolar–vascular development. Reduced expression of VEGFR and PECAM-1 has been reported in lung tissues from infants with BPD and is associated with impaired pulmonary vascular growth and alveolar simplification (Le Cras et al., 2002; Thébaud and Abman, 2007; Bolte et al., 2020). Experimental studies have demonstrated that FOXF1 promotes endothelial responsiveness to VEGF by regulating the expression of VEGFR2 (FLK1), thereby supporting angiogenic signaling during lung development (Kalinichenko et al., 2001; Ren et al., 2014).

In the context of BPD-PH, FOXF1 deficiency may contribute to disease progression by attenuating VEGF-dependent endothelial regeneration and vascular repair. Because VEGF signaling represents a downstream effector of FOXF1 activity, impaired FOXF1 expression could amplify pulmonary vascular rarefaction and disrupt alveolar–vascular coupling. Thus, FOXF1 may function as an upstream regulator that integrates multiple angiogenic pathways rather than acting solely through VEGF signaling (Kalinichenko et al., 2001; Ren et al., 2014; Ren et al., 2019; Bolte et al., 2020).

Building upon the physiological framework summarized in **Figure 1**, current evidence indicates that multiple injurious stimuli converge on FOXF1 downregulation, thereby disrupting endothelial repair, angiogenesis, epithelial differentiation, and vascular maturation. These convergent mechanisms form the disease-oriented conceptual framework illustrated in **Figure 2**.

Accordingly, **Figure 2** should be interpreted as a disease-oriented conceptual framework that integrates the mechanistic evidence reviewed in Sections 4.1–4.5 rather than depicting a single linear pathogenic pathway.

### 4.6 An integrated pathophysiological conceptual framework linking FOXF1 dysregulation to BPD-PH

As summarized in **Figure 2**, current evidence supports a unifying pathophysiological framework in which developmental lung injury converges on FOXF1 dysregulation through multiple upstream mechanisms (Hansmann et al., 2021; Acharya et al., 2023; El-Saie et al., 2023; Durlak and Thébaud, 2024; Shirazi et al., 2025). Reduced FOXF1 activity subsequently impairs endothelial progenitor cell maintenance, attenuates angiogenic signaling, disrupts vascular maturation, and alters epithelial–mesenchymal communication (Ren et al., 2014; Ren et al., 2019; Wang et al., 2022; Bian et al., 2023; Reza et al., 2023; Shirazi et al., 2025). The cumulative consequence is pulmonary vascular rarefaction, arrested alveolarization, and progressive vascular remodeling, ultimately leading to pulmonary hypertension.

This framework does not establish FOXF1 as the sole driver of BPD-PH. Rather, it positions FOXF1 as a central molecular integrator through which multiple pathogenic stimuli may converge. Future studies will be required to determine the relative contribution of FOXF1 dysregulation compared with other established pathways involved in developmental pulmonary vascular disease.

## 5 Opportunities and challenges for FOXF1-targeted prevention and therapy in BPD-PH

Restoration of FOXF1 signaling has emerged as a promising therapeutic strategy because FOXF1 occupies an upstream regulatory position within multiple developmental pathways involved in pulmonary angiogenesis, endothelial repair, and alveolar maturation (Ren et al., 2014; Ren et al., 2019; Wang et al., 2022; Reza et al., 2023). Unlike therapies targeting individual downstream mediators, FOXF1-based approaches have the potential to simultaneously influence several interconnected biological processes. Nevertheless, most available evidence remains confined to preclinical studies, and substantial challenges must be overcome before FOXF1-targeted therapies can be translated into clinical practice for premature infants with BPD-PH.

### 5.1 Nanoparticle-mediated FOXF1 gene delivery

Nanoparticle (NP)-mediated gene delivery is currently the most extensively studied therapeutic strategy for FOXF1 activation. The PEI-PEG nanoparticles (npFOXF1) developed by Kalinichenko’s team can efficiently deliver FOXF1 expression vectors to pulmonary capillary endothelial cells (Dunn et al., 2018). Bolte et al. demonstrated that combined intravenous administration of npFOXF1 and npFOXM1 significantly increased endothelial cell proliferation, promoted pulmonary angiogenesis and alveolarization, improved the elastic fiber network structure, and protected adult lung function (Bolte et al., 2020). Systematic studies in the ACDMPV model have also confirmed that nanoparticle-mediated delivery of STAT3 (a downstream effector molecule of FOXF1) prevents PH and right ventricular hypertrophy, and improves survival rates (Sun et al., 2021). Preliminary safety studies of npFOXF1 in wild-type mice showed no significant toxicity (Kohram et al., 2023), providing a safety foundation for further clinical translation.

The main advantages of the nanoparticle delivery system lie in the following: the non-integrating expression vector avoids the risk of genomic integration; relative selectivity for targeting pulmonary endothelial cells can be achieved by adjusting particle size and surface modification; and the systemic intravenous administration route makes clinical application in neonates feasible. However, clinical translation to human neonates still faces challenges such as batch-to-batch consistency, target organ selectivity, and human safety, which require further validation in large animal models.

Collectively, these studies provide proof-of-concept evidence that restoration of FOXF1 signaling can rescue developmental lung injury. However, the therapeutic efficacy observed in rodent models may not fully predict clinical performance in human neonates, particularly given differences in lung maturation, immune responses, and nanoparticle biodistribution (Dunn et al., 2018; Bolte et al., 2020; Sun et al., 2021; Kohram et al., 2023).

### 5.2 AAV-mediated targeted delivery of FOXF1

Aden-associated virus (AAV) vectors represent another important platform for FOXF1 gene therapy. Isobe et al. demonstrated that delivery of Foxf1 to EC-Bmpr2-/- mice via an AAV vector specifically targeting pulmonary vascular endothelial cells not only repaired endothelial cell DNA damage and activated angiogenesis genes (including Vegfr2, Acvrl1, Eng and Smad9), but also achieved complete reversal of experimental PAH (Isobe et al., 2023).

Compared with nanoparticle systems, the potential advantages of AAV lie in higher gene transduction efficiency and more sustained expression; the main challenges include immunogenicity, the impact of pre-existing neutralizing antibodies on delivery efficiency, and the potential risk of genomic integration (although rare) (Bulcha et al., 2021). Despite encouraging preclinical results, several translational barriers remain. Long-term transgene expression may be advantageous for chronic disease but raises concerns about developmental safety in rapidly growing lungs (Naldini, 2015; High and Roncarolo, 2019). In addition, vector immunogenicity, manufacturing complexity, and uncertainties regarding optimal dosing in premature infants remain unresolved challenges.

### 5.3 Small-molecule FOXF1 activation strategy: TanFe compound

TanFe (transcellular activator of nuclear FOXF1 expression) is a small-molecule compound identified through screening of nitrile-based compounds. It exerts its effect by inhibiting the ubiquitination and degradation of the FOXF1 protein mediated by the E3 ubiquitin ligase HECTD1, thereby increasing FOXF1 protein levels (Pradhan et al., 2023). In ACDMPV mouse models and acute lung injury models, TanFe has demonstrated pro-angiogenic activity; its vascular repair effects have also been confirmed in vascular organoids derived from human induced pluripotent stem cells (iPSCs from ACDMPV patients) (Wang et al., 2022). Administration of TanFe to pregnant mice prevented postnatal mortality in Foxf1+/- newborn mice, suggesting its potential for perinatal intervention in ACDMPV (Pradhan et al., 2023). Small-molecule drugs offer significant advantages over gene therapy vectors in the drug development process; however, their selectivity and safety in the neonatal population still require systematic evaluation.

Among currently available FOXF1-targeted approaches, small-molecule stabilization strategies may offer the most straightforward translational pathway because they avoid many of the manufacturing, delivery, and regulatory challenges associated with gene therapy. Nevertheless, the specificity of FOXF1 activation and potential off-target developmental effects require careful evaluation before clinical application.

### 5.4 The Semaphorin signaling pathway as an upstream therapeutic target

Based on recent findings that Semaphorin signaling defects in BPD-PH lead to downregulation of FOXF1, activation of the Semaphorin signaling pathway (e.g. via recombinant Semaphorin proteins or gene enhancement strategies) may offer a therapeutic approach to restore FOXF1 function at an upstream level (Shirazi et al., 2025). The development of relevant therapeutic strategies requires first elucidating the specific Semaphorin subtypes (e.g., SEMA3B, SEMA6A) and their receptor combinations that are impaired in BPD-PH, which awaits further functional mechanistic studies. Targeting Semaphorin signaling may offer a biologically attractive alternative to direct FOXF1 replacement because it aims to restore endogenous developmental signaling networks. However, the precise Semaphorin ligands, receptors, and downstream mechanisms responsible for FOXF1 regulation in BPD-PH remain incompletely defined and require further investigation.

### 5.5 Translational medical models and organoid research platforms

A major challenge in the study of developmental lung diseases is the limited availability of experimental systems capable of reproducing the complex interactions between epithelial, endothelial, and mesenchymal cell populations.

Recent advances in vascularized lung organoid technology have made it possible to partially reconstruct the developmental lung microenvironment *in vitro*. These systems provide a useful platform for studying FOXF1-related signaling pathways, endothelial dysfunction and alveolar development under controlled experimental conditions (Miao et al., 2025).

Furthermore, organoid-based models facilitate mechanistic studies of developmental signaling interactions and allow for the preliminary evaluation of candidate therapeutic strategies, including targeted delivery systems and pathway modulators. Nevertheless, current organoid systems are still unable to fully replicate the structural and physiological complexity of the developing neonatal lung.

Further refinement of these models holds promise for deepening our understanding of the pathogenesis of bronchopulmonary dysplasia with pulmonary hypertension (BPD-PH) and supporting future translational research.

### 5.6 Potential limitations of FOXF1-based therapies

Although restoration of FOXF1 signaling represents an attractive therapeutic concept, several limitations should be considered. FOXF1 is a developmental transcription factor with pleiotropic and cell-type-specific functions, raising concerns that excessive or prolonged activation may disrupt normal developmental programs (Ren et al., 2014; Ustiyan et al., 2018; Guo et al., 2023; Reza et al., 2023). Achieving cell-selective delivery and defining the optimal developmental window therefore remain major challenges.

No validated biomarker currently distinguishes therapeutic restoration of FOXF1 activity from excessive or potentially detrimental activation. Preclinical monitoring should therefore integrate molecular, developmental, and functional assessments. Candidate pharmacodynamic readouts may include restoration of FOXF1-associated signaling, such as VEGFR2/FLK1, ACVRL1, PDGFB, and WNT5A. Complementary developmental endpoints may include pulmonary capillary density, secondary septation, alveolar complexity, and endothelial progenitor cell abundance (Ren et al., 2014; Ren et al., 2019; Bolte et al., 2020; Wang et al., 2022; Kohram et al., 2023; Pradhan et al., 2023; Reza et al., 2023). Functional monitoring should incorporate pulmonary vascular remodeling, serial echocardiographic assessment of pulmonary hypertension, postnatal lung growth, and longitudinal respiratory outcomes. Together, these complementary assessments may provide a reasonable multidimensional strategy for defining the therapeutic window of FOXF1-targeted interventions.

Most available evidence is derived from rodent models, and the optimal timing, dose, and duration of FOXF1 activation have not been established (Ren et al., 2019; Bolte et al., 2020). Differences in lung development, immune maturation, and pulmonary vascular biology may limit direct translation to premature infants. Validation in large-animal models, human lung organoids, and well-characterized human tissue datasets will therefore be required before clinical application. No FOXF1-targeted therapy has yet entered clinical trials for BPD-PH.

Given the diversity of emerging FOXF1-directed therapeutic approaches, a comparative evaluation of their mechanisms, experimental evidence, translational advantages, and current limitations is warranted. The major FOXF1-targeted therapeutic strategies currently under investigation are summarized in **Table 3**.

**Table 3:** Emerging FOXF1-targeted therapeutic strategies for bronchopulmonary dysplasia-associated pulmonary hypertension (BPD-PH).

Therapeutic strategy	Mechanism of action	Experimental evidence	Potential advantages	Current limitations	Translational readiness
Nanoparticle-mediated FOXF1 gene delivery (npFOXF1)	Delivers FOXF1 expression constructs to pulmonary endothelial cells, restoring FOXF1-dependent angiogenic and regenerative signaling	Hyperoxia-induced BPD models and FOXF1-deficient developmental lung injury models demonstrated improved angiogenesis, alveolarization, and pulmonary vascular development (Dunn et al., 2018; Bolte et al., 2020; Kohram et al., 2023)	Non-integrating platform; endothelial targeting capability; simultaneous restoration of multiple downstream pathways	No human studies; biodistribution and long-term safety remain uncertain; delivery efficiency requires optimization	**Moderate**
AAV-mediated FOXF1 gene therapy	Sustained endothelial FOXF1 expression through adeno-associated viral vector delivery	Experimental pulmonary hypertension models showed restoration of endothelial function and angiogenic signaling (Isobe et al., 2023)	High transduction efficiency; durable gene expression; strong preclinical efficacy	Potential immunogenicity; manufacturing complexity; uncertain developmental safety in premature infants	**Moderate**
Small-molecule FOXF1 stabilizer (TanFe)	Prevents HECTD1-mediated FOXF1 degradation, increasing endogenous FOXF1 protein stability and activity	Improved angiogenesis and vascular development in ACDMPV mouse models and human iPSC-derived vascular organoids (Pradhan et al., 2023)	Easier manufacturing and clinical translation than gene therapy; potentially scalable treatment approach	Specificity and off-target developmental effects remain incompletely characterized	**Moderate–High**
Semaphorin pathway modulation	Restores endogenous Semaphorin–FOXF1 signaling and endothelial–epithelial communication	Supported by human single-cell and spatial transcriptomic analyses of BPD-PH and ACDMPV tissues (Shirazi et al., 2025)	Targets upstream developmental signaling network; may restore physiological FOXF1 regulation	Therapeutic agents not yet established; causal mechanisms require further validation	**Early-stage**
Human vascularized lung organoids	Human-relevant platform for modeling FOXF1 signaling and evaluating therapeutic responses	Successfully used to investigate FOXF1-dependent vascular developmental pathways and therapeutic candidates (Miao et al., 2025)	Enables patient-specific disease modeling; supports precision medicine and preclinical screening	Experimental platform rather than direct therapy; limited ability to fully recapitulate neonatal lung physiology	**Research platform**

Bold values indicate the translational readiness level of each therapeutic strategy.

### 5.7 Summary of translational potential

Taken together, current evidence suggests that FOXF1 is one of the most promising developmental regulators currently under investigation for BPD-PH. Gene-delivery platforms, small-molecule FOXF1 stabilizers, and upstream pathway modulation strategies have each demonstrated encouraging preclinical efficacy (Bolte et al., 2020; Sun et al., 2021; Isobe et al., 2023; Pradhan et al., 2023). However, substantial challenges related to safety, cell-specific targeting, developmental timing, and clinical feasibility remain unresolved. Future translational efforts should focus on defining optimal intervention windows and validating therapeutic efficacy in human-relevant experimental systems.

## 6 Future directions and outstanding questions

Despite substantial progress in understanding FOXF1 biology, several fundamental questions remain unresolved regarding its role in BPD-PH. Although current evidence supports FOXF1 as an important regulator of pulmonary vascular and alveolar development, the precise mechanisms through which FOXF1 contributes to human disease pathogenesis remain incompletely understood. Addressing these knowledge gaps will be critical for translating FOXF1-based discoveries into clinically meaningful applications.

### 6.1 Is FOXF1 a causal driver of human BPD-PH?

A central unresolved question is whether FOXF1 dysregulation directly drives disease progression or primarily reflects ongoing developmental lung injury. Experimental studies have shown that FOXF1 deficiency impairs endothelial progenitor cell maintenance, pulmonary angiogenesis, and alveolar development, whereas restoration of FOXF1 signaling improves lung structure and vascular growth in preclinical models (Ren et al., 2014; Dunn et al., 2018; Ren et al., 2019; Bolte et al., 2020; Sun et al., 2021; Wang et al., 2022; Isobe et al., 2023; Kohram et al., 2023). However, most available evidence remains derived from animal models and *in vitro* systems.

Recent human single-cell and spatial transcriptomic studies have identified reduced FOXF1 expression and altered FOXF1-associated signaling networks in BPD-PH lungs (Shirazi et al., 2025). Nevertheless, these observations are largely correlative. Future investigations integrating human tissue datasets, longitudinal clinical samples, and mechanistic studies will be necessary to determine whether FOXF1 functions as a primary pathogenic driver, a disease modifier, or a downstream consequence of developmental lung injury.

### 6.2 How does FOXF1 coordinate alveolar–vascular development across different cell types?

Although FOXF1 is best known for its role in endothelial biology, accumulating evidence indicates that its regulatory functions extend to mesenchymal and epithelial compartments (Ustiyan et al., 2018; Guo et al., 2023; Reza et al., 2023). Future studies should clarify how FOXF1 coordinates signaling among different cell populations during lung development and injury repair.

Advanced approaches such as single-cell multi-omics, spatial transcriptomics, lineage-tracing strategies, and vascularized lung organoid systems may help define cell-type-specific FOXF1 functions and identify the compartments most relevant to disease progression and therapeutic targeting (Guo et al., 2023; Miao et al., 2025; Shirazi et al., 2025).

### 6.3 Can FOXF1-targeted therapies be successfully translated to premature infants?

Restoration of FOXF1 activity through nanoparticle-mediated gene delivery, AAV-based gene therapy, small-molecule FOXF1 stabilizers, and upstream pathway modulation has shown encouraging results in preclinical models (Dunn et al., 2018; Bolte et al., 2020; Sun et al., 2021; Isobe et al., 2023; Kohram et al., 2023; Pradhan et al., 2023). However, substantial translational challenges remain before these approaches can be applied clinically.

Key unresolved issues include identification of the optimal therapeutic window, establishment of long-term safety profiles, achievement of cell-specific targeting, and validation of efficacy in human-relevant experimental systems. Because the premature lung exists within a rapidly changing developmental environment, treatment responsiveness may differ substantially from that observed in adult disease models. Future studies using large-animal models, vascularized human lung organoids, and integrated multi-omics datasets will be essential to bridge the gap between experimental efficacy and clinical application (Guo et al., 2023; Miao et al., 2025; Shirazi et al., 2025).

Collectively, these outstanding questions highlight the need for a more comprehensive understanding of FOXF1 biology across developmental, pathological, and therapeutic contexts. Continued advances in multi-omics technologies, gene-editing approaches, and humanized disease models provide unprecedented opportunities to address these challenges and further define the role of FOXF1 in BPD-PH.

## 7 Conclusion

Bronchopulmonary dysplasia-associated pulmonary hypertension (BPD-PH) remains one of the most serious complications of extreme prematurity and is characterized by the combined presence of arrested alveolarization, pulmonary vascular rarefaction, and progressive vascular remodeling. Increasing evidence suggests that these pathological processes are not independent events but rather interconnected manifestations of disrupted alveolar–vascular development.

Recent advances in developmental biology, single-cell transcriptomics, spatial omics, and experimental disease models have identified FOXF1 as a central regulator of pulmonary vascular growth, endothelial progenitor cell maintenance, vascular maturation, and alveolar development. Through coordinated regulation of VEGF/FLK1, BMP9/ACVRL1, PDGF/TIE2, Notch, WNT5A, and Semaphorin-associated signaling networks, FOXF1 occupies a strategic position at the interface between vascular and alveolar development.

Based on current evidence, we propose a conceptual framework in which FOXF1 functions as a molecular nexus linking arrested alveolarization to pulmonary vascular remodeling in BPD-PH. Reduced FOXF1 activity may impair endothelial regeneration, disrupt epithelial–mesenchymal communication, attenuate angiogenic signaling, and promote pathological vascular remodeling, collectively contributing to disease progression.

Importantly, available data remain derived predominantly from animal models and observational human studies. Therefore, the precise causal role of FOXF1 in human BPD-PH, its cell-type-specific functions, and its therapeutic potential require further validation. Nevertheless, emerging strategies including nanoparticle-mediated gene delivery, AAV-based gene therapy, small-molecule FOXF1 stabilizers, and advanced human lung organoid systems have created new opportunities for translational investigation. Importantly, the current evidence base remains heavily weighted toward mechanistic and preclinical studies, whereas direct clinical evidence in infants with BPD-PH is currently lacking. Therefore, FOXF1 should be considered a promising investigational target rather than an established therapeutic pathway.

In summary, FOXF1 represents a promising developmental regulator implicated in the pathobiology of BPD-PH. By coordinating endothelial progenitor cell maintenance, angiogenic signaling, vascular maturation, epithelial–mesenchymal communication, and alveolar development, FOXF1 may provide a conceptual link between arrested alveolarization and pulmonary vascular remodeling. However, FOXF1 should currently be regarded as an investigational biomarker and therapeutic target rather than an established clinical pathway. Future studies integrating human multi-omics datasets, vascularized lung organoids, and translational animal models will be essential to determine its causal role and therapeutic feasibility in infants with BPD-PH.
